# Short-lived long noncoding RNAs as surrogate indicators for chemical stress in HepG2 cells and their degradation by nuclear RNases

**DOI:** 10.1038/s41598-019-56869-y

**Published:** 2019-12-30

**Authors:** Hidenori Tani, Ayaka Numajiri, Motohide Aoki, Tomonari Umemura, Tetsuya Nakazato

**Affiliations:** 1 Environmental Management Research Institute, National Institute of Advanced Industrial Science and Technology (AIST), 16-1, Onogawa, Tsukuba, Ibaraki 305-8569 Japan; 2 Department of Molecular Life Sciences, School of Life Sciences, Tokyo University of Pharmacy and Life Sciences, 1432-1 Horinouchi, Hachioji, Tokyo 192-0392 Japan

**Keywords:** Long non-coding RNAs, RNA decay

## Abstract

Long noncoding RNAs (lncRNAs) are non-protein-coding transcripts >200 nucleotides in length that have been shown to play important roles in various biological processes. The mechanisms underlying the induction of lncRNA expression by chemical exposure remain to be determined. We identified a novel class of short-lived lncRNAs with half-lives (*t*_1/2_) ≤4 hours in human HeLa Tet-off cells, which have been suggested to express many lncRNAs with regulatory functions. As they may affect various human biological processes, short-lived lncRNAs may be useful indicators of the degree of stress on chemical exposure. In the present study, we identified four short-lived lncRNAs, designated as OIP5-AS1, FLJ46906, LINC01137, and GABPB1-AS1, which showed significantly upregulated expression following exposure to hydrogen peroxide (oxidative stress), mercury II chloride (heavy metal stress), and etoposide (DNA damage stress) in human HepG2 cells. These lncRNAs may be useful indicators of chemical stress responses. The levels of these lncRNAs in the cells were increased because of chemical stress-induced prolongation of their decay. These lncRNAs were degraded by nuclear RNases, which are components of the exosome and XRN2, and chemical exposure inhibited the RNase activities within the cells.

## Introduction

Large numbers of intergenic, intronic, and cis-antisense long noncoding RNAs (lncRNAs), non-protein-coding transcripts >200 nucleotides in length, have been identified in mammals and shown to be transcribed in a tissue- and developmental stage-specific manner^1,2^. These lncRNAs play a wide range of structural and regulatory roles in various cellular processes, including chromatin remodeling, imprinting, transcription, translation, and epigenetic regulation^3,4^.

For example, the lncRNA, HOX transcript antisense RNA (HOTAIR), has been reported to play roles in the regulation of gene expression via a mechanism involving epigenetic modification^5^. HOTAIR interacts with the catalytic subunit of polycomb repressive complex 2 (PRC2) and enhancer of zeste homolog 2 (EZH2) via its 5′ end^6,7^, while the 3′ end interacts with the histone demethylase lysine-specific histone demethylase 1 A (LSD1)^7^. Another lncRNA, nuclear-enriched abundant transcript 1 (NEAT1), has been shown to an essential structural component of paraspeckles^8–10^. The interaction of NEAT1 with splicing factor proline- and glutamine-rich (SFPQ) was shown to be involved in both the repression and activation of gene expression in a manner depending on the context of the promoter sequence and interactions with other transcription factors^11–14^. The lncRNA, Growth arrest-specific 5 (GAS5), is upregulated under conditions of serum starvation or treatment with translation inhibitors that induce growth arrest^15,16^, and has been shown to function as a riborepressor for the glucocorticoid receptor (GR) by binding to its DNA-binding domain and acting as a decoy glucocorticoid response element (GRE)^17^. The regulatory role of GAS5 for the apoptosis-related genes, cellular inhibitor of apoptosis protein 1 (cIAP2) and serum/glucocorticoid regulated kinase 1 (SGK1), is controlled by its RNA degradation pathway^18^. Although previous studies have demonstrated a number of important roles of lncRNAs, little is known regarding changes in their expression levels on exposure to chemical stressors.

The half-lives of lncRNAs were reported to show a wide range of variation, comparable to those of mRNAs in mouse neuro-2a neuroblastoma cells and human HeLa Tet-off cells^19,20^. Studies using the 5′-bromouridine immunoprecipitation chase-deep sequencing (BRIC-seq) genome-wide method for determining RNA stability^20–23^ showed that well-known regulatory lncRNAs, such as HOTAIR, NEAT1, and GAS5, have short half-lives (*t*_1/2_ ≤ 4 hours), while housekeeping lncRNAs, such as rRNAs, small nucleolar RNAs (snoRNAs), and small Cajal body-specific RNAs (SCARNAs), have long half-lives (*t*_1/2_ > 4 hours)^20^. These observations suggest that the half-lives of lncRNAs are correlated with their functional characteristics, and that the short-lived lncRNAs may include a disproportionate number of those with regulatory functions. Therefore, as yet unidentified short-lived lncRNAs may affect biological processes in humans, and they may therefore be useful as indicators of the degree of chemical stress in cells^24^.

The present study was performed to identify novel short-lived lncRNAs that show changes in expression on exposure to chemical stressors in HepG2 cells. This cell line is a useful *in vitro* model of human chemical detoxification, is easy to handle, and provides a reproducible human model system. Four lncRNAs, designated as OIP5-AS1, FLJ46906, LINC01137, and GABPB1-AS1, which showed upregulation of expression in response to chemical stressors, were identified in HepG2 cells and these lncRNAs were shown to be degraded by nuclear RNases.

## Results

### Screening for lncRNAs showing chemical stress-induced expression changes

Twenty-six lncRNAs with short half-lives (*t*_1/2_ ≤ 4 hours) in HeLa Tet-off cells were first selected according to the following criteria^20^: (1) >200 nt; (2) highly and moderately expressed transcripts with RPKM (reads per kilobase million) >1 on RNA-Seq analysis in HeLa Tet-off cells; (3) searchable on NCBI; (4) no overlap with regions containing known protein-coding genes; and (5) excluding well-known short-lived lncRNAs, such as HOTAIR, NEAT1, and GAS5 (Table 1). HepG2 cells were treated with hydrogen peroxide (oxidative stress), mercury II chloride (heavy metal stress), and etoposide (DNA damage stress) at a concentration of 100 μM for 24 hours as described previously^25^ as chemical stressors, and alterations in the expression levels of the 26 selected lncRNAs were examined.

**Table 1:** The short-lived lncRNAs investigated in the present study.

Name	Accession No.	Length (nt)	*t*_1/2_^*^ (h)
MIR22HG	NR_028502	2,699	2.4
LINC-PINT	NR_015431	2,964	2.4
KMT2E-AS1	NR_024586	3,615	2.3
LINC00667	NR_015389	3,979	3.4
HCG18	NR_024052	6,814	2.9
UBA6-AS1	NR_015439	2,301	3.2
LINC00662	NR_027301	2,085	2.7
GABPB1-AS1	NR_024490	4,139	3.4
LINC01184	NR_015360	2,977	3.5
TTN-AS1	NR_038271	2,033	2.6
LINC00473_v1	NR_026860	1,832	2.4
LINC00473_v2	NR_026861	1,123	2.4
FAM222A-AS1	NR_026661	1,178	2.4
CYTOR	NR_024204	828	2.4
MIR4435-2HG_v1	NR_015395	809	2.5
MIR4435-2HG_v2	NR_024373	557	2.3
IDI2-AS1	NR_024628	1,107	3.7
SNHG15	NR_003697	837	2.6
ZFP91-CNTF	NR_024091	3,544	3.6
OIP5-AS1	NR_026757	5,320	3.4
KMT2E-AS1	NR_024586	3,600	3.4
EBLN3P	NR_036592	2,265	3.4
LINC01137	NR_038842	997	3.0
PVT1	NR_003367	1,918	2.4
FLJ46906	NR_033896	1,022	2.7
PP7080	NR_024158	1,760	2.8

^*^Values are from Tani *et al*.^20^.

**Figure 1:**
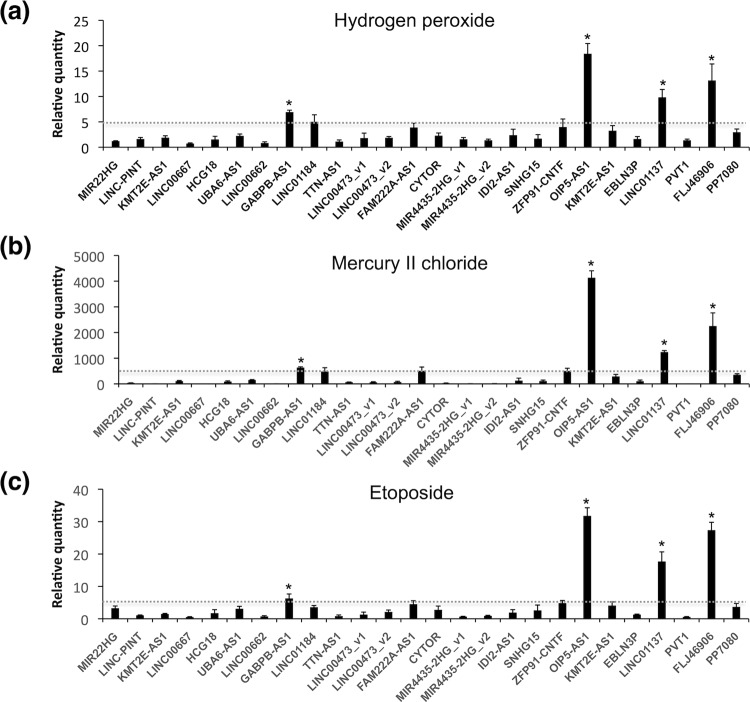
Exposure to chemical stressors altered the levels of lncRNA expression. Following treatment of HepG2 cells with 100 μM (**a**) hydrogen peroxide, (**b**) mercury II chloride, or (**c**) etoposide for 24 hours, RT-qPCR was performed to determine the expression levels of the indicated RNAs normalized relative to GAPDH, ACTB, HPRT1, and PGK1. All values are means ± SD from four independent experiments (*P < 0.05, Student’s *t* test). The expression levels of treated cells divided by those of untreated cells are shown on y-axis, so a value of 0 indicates that the RNA could not be detected, while a value of 1 indicates no change in expression level compared to untreated controls. Gray dotted lines indicated cut-off values.

Differences in expression could be measured easily for lncRNAs that showed marked and rapid upregulation on exposure to chemical stressors. Therefore, we focused on lncRNAs that were upregulated in response to chemical exposure as potential chemical stress indicators. Significant increases of 5-, 500-, and 5-fold in the expression levels of OIP5 antisense RNA 1 (OIP5-AS1), FLJ46906, long intergenic non-protein-coding RNA 1137 (LINC01137), and GABPB1 antisense RNA 1 (GABPB1-AS1) were observed following treatment with 100 μM hydrogen peroxide, 100 μM mercury II chloride, or 100 μM etoposide, respectively (Fig. 1). Although lncRNAs showing downregulated expression in response to chemical exposure are also potentially important, the greatest degree of downregulation was observed for LINC00667 in cells treated with 100 μM etoposide, but the degree of change was only 0.53-fold (Fig. 1c). Therefore, downregulated lncRNAs were not included in further analysis.

**Figure 2:**
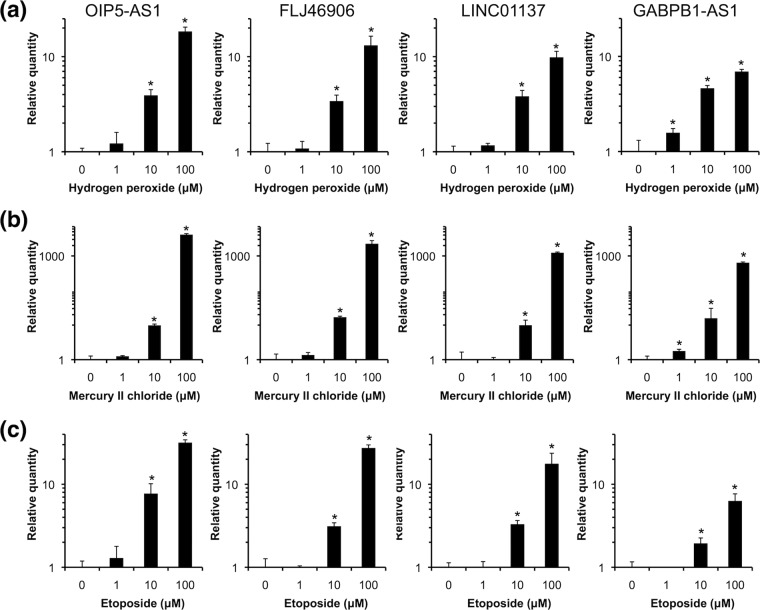
Chemical stressors changed lncRNA expression levels. Following treatment of HepG2 cells with 100 μM (**a**) hydrogen peroxide, (**b**) mercury II chloride, or (**c**) etoposide for 24 hours, RT-qPCR was performed to determine the expression levels of the indicated RNAs normalized relative to GAPDH, ACTB, HPRT1, and PGK1. All values are means ± SD from four independent experiments (*P < 0.05, Student’s *t* test).

The degrees of change in expression of the four lncRNAs upregulated following treatment with hydrogen peroxide, mercury II chloride, and etoposide at various concentrations, i.e., OIP5-AS1, FLJ46906, LINC01137, and GABPB1-AS1, were examined (Fig. 2). The levels of OIP5-AS1, FLJ46906, LINC01137, and GABPB1-AS1 expression increased with increasing hydrogen peroxide, mercury II chloride, and etoposide concentrations, indicating a dose-dependent response to chemical stressors. Therefore, monitoring the expression levels of these lncRNAs may be useful as indicators of the degree of chemical stress in HepG2 cells.

### Exposure to chemical stressors reduced the rates of decay of lncRNAs

**Figure 3:**
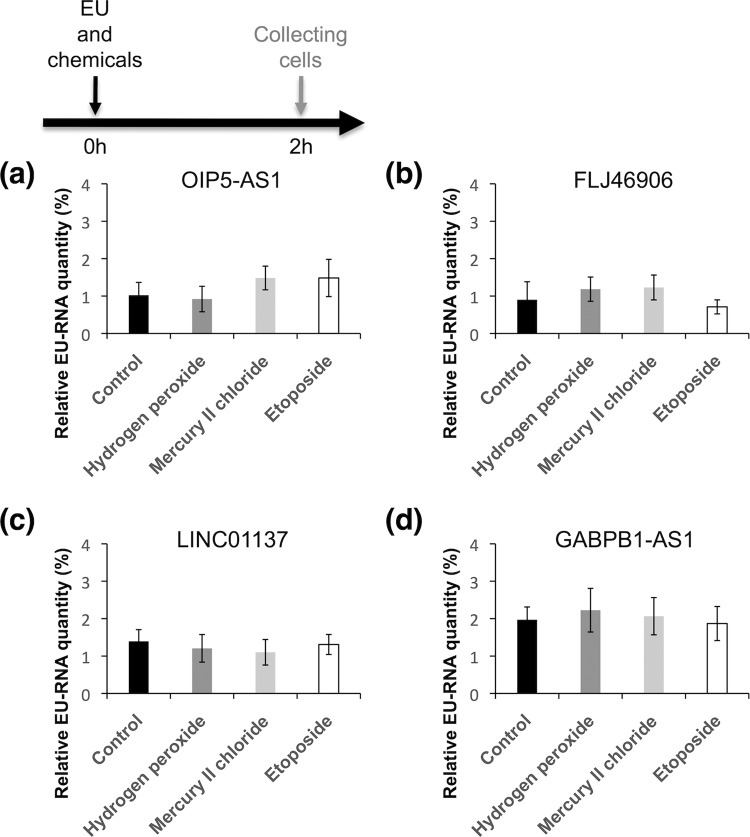
The lncRNA transcription rates were not affected by chemical stressors. The transcription rates of the lncRNAs (**a**) OIP5-AS1, (**b**) FLJ46906, (**c**) LINC00137, and (**d**) GABPB1-AS1 were examined in control cells (black bar) and those exposed to hydrogen peroxide (gray bar), mercury II chloride (pale gray bar), or etoposide (white bar) as chemical stressors. The nascent lncRNAs incorporated EU during transcription, and the relative EU-RNA quantity reflects the total amount of EU-labeled RNA captured divided by the input amount of RNA as an indicator of the transcription rate. All values are means ± SD from three independent experiments.

**Figure 4:**
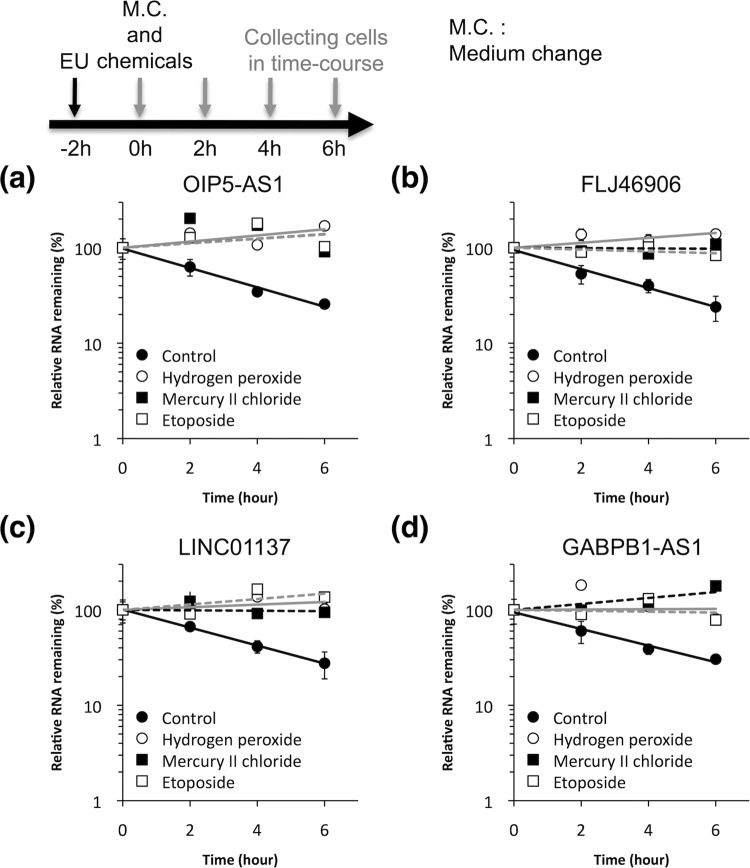
The decay rates of lncRNAs were reduced by chemical stressors. The rates of decay of the lncRNAs (**a**) OIP5-AS1, (**b**) FLJ46906, (**c)** LINC00137, and (**d**) GABPB1-AS1 were examined in control cells (solid circles and black lines) and those exposed to hydrogen peroxide (open circles/gray line), mercury II chloride (solid squares/black dotted line), or etoposide (open squares/gray dotted line) as chemical stressors. All values are means ± SD from three independent experiments.

Next, we examined whether the increased levels of expression of the four lncRNAs, OIP5-AS1, FLJ46906, LINC01137, and GABPB1-AS1, under conditions of chemical stress (i.e., exposure to hydrogen peroxide, mercury II chloride, and etoposide) were due to increases in their transcription rates or decreases in their decay rates by determining their transcription rates and half-lives using the 5-ethynyluridine (EU) pulse labeling method^25–28^ (Figs. 3 and 4). This method involves the separation of EU-labeled RNAs (EU-RNAs) from total RNAs by biotinylation of EU in a copper-catalyzed cycloaddition reaction, followed by purification using streptavidin-conjugated magnetic beads. The rates of transcription were analyzed by incubating cells in culture medium with addition of EU and chemical stressors for 2 hours, followed by measuring the levels of EU-labeled RNA by quantitative reverse transcription PCR (RT-qPCR). Cells treated with 100 μM hydrogen peroxide, 100 μM mercury II chloride, or 100 μM etoposide showed no significant differences in transcription rates of the four lncRNAs compared to the controls (Fig. 3).

Assessment of the half-lives of the four lncRNAs involved incubation of the cells in culture medium containing EU for 2 hours, and isolation of total RNAs including EU-RNAs at various time points after the removal of surplus EU from the culture medium along with the addition of chemical stressors. The levels of EU-RNAs were then quantified by RT-qPCR. Treatment of the cells with each of the chemical stressors (i.e., 100 μM hydrogen peroxide, 100 μM mercury II chloride, or 100 μM etoposide) resulted in increases in the *t*_1/2_ of OIP5-AS1 from 2.8 hours to >6 hours (Fig. 4a), that of FLJ46906 from 2.4 hours to >6 hours (Fig. 4b), that of LINC01137 from 3.2 hours to >6 hours (Fig. 4c), and that of GABPB1-AS1 from 2.4 hours to >6 hours (Fig. 4D). Therefore, the increases in levels of these four lncRNAs in response to chemical exposure were due to reduced rates of decay rather than to increased rates of transcription.

### Knockdown of nuclear RNase and helicase expression levels

**Figure 5:**
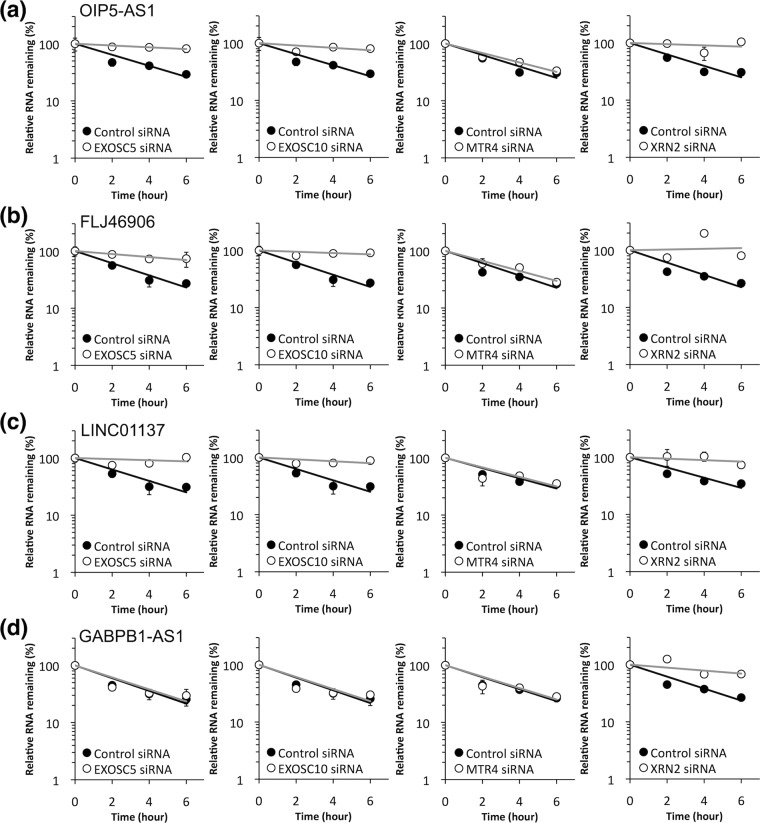
Nuclear RNase and helicase knockdown experiments. The rates of decay of the lncRNAs (**a**) OIP5-AS1, (**b**) FLJ46906, (**c**) LINC00137, and (**d**) GABPB1-AS1 were examined in control cells transfected with a control siRNA expression vector (solid circles/black line) and those transfected with siRNAs targeting the indicated nuclear RNases or helicase (open circles/gray line). All values are means ± SD from three independent experiments.

The factors contributing to the degradation of the four lncRNAs, OIP5-AS1, FLJ46906, LINC01137, and GABPB1-AS1, were next examined in HepG2 cells. As these four lncRNAs are localized in the nucleus^20^, we examined the contributions to their nuclear degradation of the following nuclear components: exosome component 5 (EXOSC5) and exosome component 10 (EXOSC10), which are core (EXOSC5) or sub- (EXOSC10) components of the RNA exosome 3′-to-5′ exonuclease complex that plays a crucial role in the degradation of RNA in eukaryotic nuclei^29,30^ along with the trimeric nuclear exosome targeting (NEXT) subunit that directs a subset of noncoding short-lived RNAs for exosomal degradation^31^ and the trimeric poly(A) tail exosome targeting (PAXT) subunit that directs a subset of long and polyadenylated poly(A) RNAs for exosomal degradation^32^; MTR4, an ATP-dependent RNA helicase, which is a cofactor for the exosome complex involved in linking to RNA-binding protein adapters^33^. The EXOSC5, EXOSC10 and MTR4 are associated with NEXT or PAXT complexs. XRN2, a nuclear 5′-to-3′ exonuclease^34^ that is involved in the torpedo model of transcription termination^35^. HepG2 cells were transfected with siRNAs specific to each of these components and examined using the 5,6-dichloro-1-β-d-ribofuranosylbenzimidazole (DRB) chase method to determine the decay rates of the lncRNAs, OIP5-AS1, FLJ46906, LINC01137, and GABPB1-AS1^28^ (Fig. 5). The levels of EXOSC5, EXOSC10, MTR4, and XRN2 transcripts in these siRNA-transfected cells were 5%, 24%, 28%, and 27%, respectively, compared to controls. The siRNA-mediated knockdown of EXOSC5, EXOSC10, and XRN2 expression increased the half-lives of three of the four lncRNAs, OIP5-AS1, FLJ46906, and LINC01137 (Fig. 5A–C), while only XRN2 knockdown increased the half-life of GABPB1-AS1 (Fig. 5D). The siRNA-mediated knockdown of MTR4 had no effect on the decay rates of the four lncRNAs (Fig. 5). As shown in Fig. S1, two different siRNAs were used in the knockdown experiments to reduce the risk of off-target effects, and the results showed that OIP5-AS1, FLJ46906, and LINC01137 were degraded by EXOSC5, EXOSC10, and XRN2, while GABPB1-AS1 was degraded only by XRN2. The small degrees of change in levels of the target RNAs were considered to have been due to low RNA knockdown efficiency, because the knockdown of XRN2 reduced the levels of the transcripts to 27%.

## Discussion

Four short-lived lncRNAs, designated as OIP5-AS1, FLJ46906, LINC01137, and GABPB1-AS1, showed changes in expression in response to chemical stress in HepG2 cells (Figs. 1 and 2). The expression levels of these lncRNAs were upregulated by 500-fold in cells exposed to heavy metal stress by treatment with mercury II chloride. This degree of upregulation was markedly greater than those observed in response to oxidative stress induced by hydrogen peroxide and DNA damage stress induced by etoposide, suggesting that mercury II chloride may have high toxicity (i.e., marked disruption of RNase function) in HepG2 cells. Previous studies have shown that short-lived lncRNAs exhibit stronger and more rapid responses to chemical stresses than traditional mRNA biomarkers^25,36^. Thus, the mRNAs biomarkers, which are undoubtedly important, were omitted in this study. Taken together with the results of previous studies indicating that the expression level of GABPB1-AS1 is commonly upregulated by chemicals in HeLa Tet-off cells and human-induced pluripotent stem cells (hiPSCs)^36,37^, our observations suggest that these four lncRNAs may be useful as indicators of common cell stress in HepG2 cells. Indeed, HepG2 cells showed stronger responses to chemical insults than HeLa Tet-off cells and hiPSCs in the present study. In addition, the findings reported here indicated that these lncRNAs are degraded by nuclear RNases, i.e., exosome components and XRN2, and that the chemical stressors examined inhibited the activities of these RNases thus preventing degradation of the lncRNAs. The results of knockdown experiments in the present study indicated that GABPB1-AS1 was only degraded by XRN2, and not affected by components of the exosome, which explained its lower degree of upregulation compared to the other three lncRNAs. To our knowledge, this study is the first to show that nuclear RNases are responsible for the degradation of chemical stress-sensitive lncRNAs.

Several recent studies showed that OIP5-AS1 suppresses cell proliferation in human cells. The RNA-binding protein, Hu Antigen R (HuR), which enhances cell proliferation, was found to be associated with OIP5-AS1 and to stabilize protein-coding mRNAs, such as cyclin A2^38^. OIP5-AS1 was also shown to interact with miR-424 and compete with HuR^38^. OIP5-AS1 was reported to reduce the mRNA expression level of cyclin-G-associated kinase (GAK), which is important for mitotic progression^39^. Moreover, OIP5-AS1 was shown to promote G_2_/M phase progression^40^. Thus, the chemical-induced upregulation of OIP5-AS1 in the present study may have been because OIP5-AS1 acted as a defense mechanism against chemical stress. Although the functions of the other three lncRNAs identified in the present study, FLJ46906, LINC01137, and GABPB1-AS1, have yet to be determined, our results indicated that the lncRNA degradation pathway plays an important role in regulating their levels in response to cellular stress. Further studies are required to elucidate the physiological functions of these lncRNAs.

Pathogenic infections represent other types of cellular stress^12,41^, and NEAT1 expression was reported to be induced in HeLa cells by influenza virus or herpes simplex virus infection. This resulted in excess paraspeckle formation and relocalization of the repressor of interleukin-8 (IL-8), SFPQ, from the promoter of IL-8 to the paraspeckles, leading to its transcriptional activation^12^. In addition, infection of HeLa cells with *Salmonella* was shown to markedly reduce the levels of the exosome/NEXT components, thus resulting in stabilization of the NEAT1 transcript, contributing to the expression of cellular immunity-related genes as part of the cellular defense mechanism against infection^41^.

The findings of this study will be useful in relating digital genomic information to cellular function, and further studies based on our results will elucidate the relationships between the novel lncRNAs identified here and RNA-binding proteins.

## Materials and Methods

### Chemicals

Hydrogen peroxide and mercury II chloride were purchased from Wako (Osaka, Japan). Etoposide was purchased from BioVision (Mountain View, CA). Both of these chemicals were dissolved in dimethyl sulfoxide (DMSO; Wako) and diluted in culture medium at a final concentration of 0.1% vol/vol.

### Cell culture

HepG2 cells were grown in Dulbecco’s modified Eagle’s medium (DMEM) supplemented with 10% fetal bovine serum (FBS) at 37 °C in a humidified incubator under an atmosphere of 5% CO_2_.

### Reverse transcription-quantitative real-time polymerase chain reaction (RT-qPCR)

Total RNA was extracted from cells with RNAiso Plus (TaKaRa, Kyoto, Japan), in accordance with the manufacturer’s instructions. The isolated RNA was reverse transcribed into cDNA using PrimeScript RT Master Mix (Perfect Real Time; TaKaRa). The resulting cDNA was amplified using the primer sets listed in Table S1, and the levels were normalized relative to glyceraldehyde-3-phosphate dehydrogenase (GAPDH), β-actin (ACTB), hypoxanthine phosphoribosyltransferase 1 (HPRT1), and phosphoglycerate kinase 1 (PGK1) mRNAs. Relative RNA quantities were calculated as treated values normalized relative to untreated values. THUNDERBIRD SYBR qPCR mix (Toyobo, Osaka, Japan) was used in accordance with the manufacturer’s instructions. RT-qPCR analysis was performed using a MyiQ2 Two-Color Real-Time PCR Detection System (Bio-Rad, Hercules, CA).

### 5-Ethynyluridine (EU) pulse labeling

Analysis of RNA transcription and degradation rates was performed by EU pulse-labeling of RNA using a Click-iT Nascent RNA Capture kit (Thermo Fisher Scientific, Wilmington, DE), in accordance with the manufacturer’s instructions, with some modifications as described previously^25–28^. To assess transcription rates, EU (400 μM) and the chemicals of interest were added to the culture medium and incubated for 2 hours. The cells were then harvested, and total RNA was isolated using RNAiso Plus (TaKaRa). EU-labeled RNAs were biotinylated and captured using a Click-iT Nascent RNA Capture kit (Thermo Fisher Scientific). To elute the EU-RNAs, magnetic beads were resuspended in 100 μl of buffer A (10 mM Tris-HCl, pH 7.4, and 6.25 mM EDTA). ISOGEN LS (Nippon Gene, Toyama, Japan) was added to the mixture, and EU-labeled RNAs were isolated in accordance with the manufacturer’s instructions; these isolated RNAs were used in subsequent RT-qPCR assays. The total amount of EU-labeled RNA captured was divided by the amount of EU-RNA input. To assess degradation rates, we added EU (200 μM) to the culture medium and incubated the cells for 2 hours. The EU-containing medium was then replaced with EU-free medium containing the chemicals of interest, and cells were harvested at the indicated time points. Total RNA was isolated using RNAiso Plus (TaKaRa). EU-labeled RNAs were biotinylated and captured using a Click-iT Nascent RNA Capture Kit (Thermo Fisher Scientific). To elute the EU-RNAs, magnetic beads were resuspended in 100 μl of buffer A (10 mM Tris-HCl, pH 7.4, and 6.25 mM EDTA). ISOGEN LS (300 μl; Nippon Gene) was added to the mixture, and EU-labeled RNAs were isolated in accordance with the manufacturer’s instructions; these isolated RNAs were used in the subsequent RT-qPCR assays.

### siRNA treatments

The sequences of the siRNAs used are listed in Table S2. These siRNAs were transfected into cells using Lipofectamine RNAiMAX (Thermo Fisher Scientific), in accordance with the manufacturer’s instructions. Briefly, siRNA duplexes were used at a final concentration of 10 nM. Cells were harvested 48 hours after transfection, and total RNAs were isolated using RNAiso Plus (TaKaRa), in accordance with the manufacturer’s instructions. RT-qPCR analysis was performed to determine whether RNA interference achieved significant depletion of each target sequence.

### DRB chase

Analysis of RNA degradation rates in siRNA treatments was performed by the 5,6-dichloro-1-β-d-ribofuranosylbenzimidazole (DRB; Sigma-Aldrich, St. Louis, MO) chase method^28^. Total RNAs were isolated in accordance with the manufacturer’s instructions at the indicated time points after addition of 20 μg/mL DRB; these isolated RNAs were used in the subsequent RT-qPCR assays.

## Supplementary information

Supplementary information.
