# Supplementary material for: Short-lived long noncoding RNAs as surrogate indicators for chemical stress in HepG2 cells and their degradation by nuclear RNases
Source: Sci Rep. 2019 Dec 30;9:20299. doi: 10.1038/s41598-019-56869-y (PMC6937343; doi:10.1038/s41598-019-56869-y)
Supplement: Supplementary file 1 — Supplementary information. [file 41598_2019_56869_MOESM1_ESM.pdf]

Supplementary information for

Short-lived long noncoding RNAs as surrogate indicators for chemical stress in HepG2 cells and their degradation by nuclear RNases

Hidenori Tani[1,*], Ayaka Numajiri[1,2], Motohide Aoki[2], Tomonari Umemura[2], Tetsuya Nakazato[1]

[1]Environmental Management Research Institute, National Institute of Advanced Industrial Science and Technology (AIST), 16-1, Onogawa, Tsukuba, Ibaraki 305-8569, Japan

[2]Department of Molecular Life Sciences, School of Life Sciences, Tokyo University of Pharmacy and Life Sciences, 1432-1 Horinouchi, Hachioji, Tokyo 192-0392, Japan

Correspondence and requests for materials should be addressed to H.T. (e-mail: h.tani@aist.go.jp)

Fig. S1

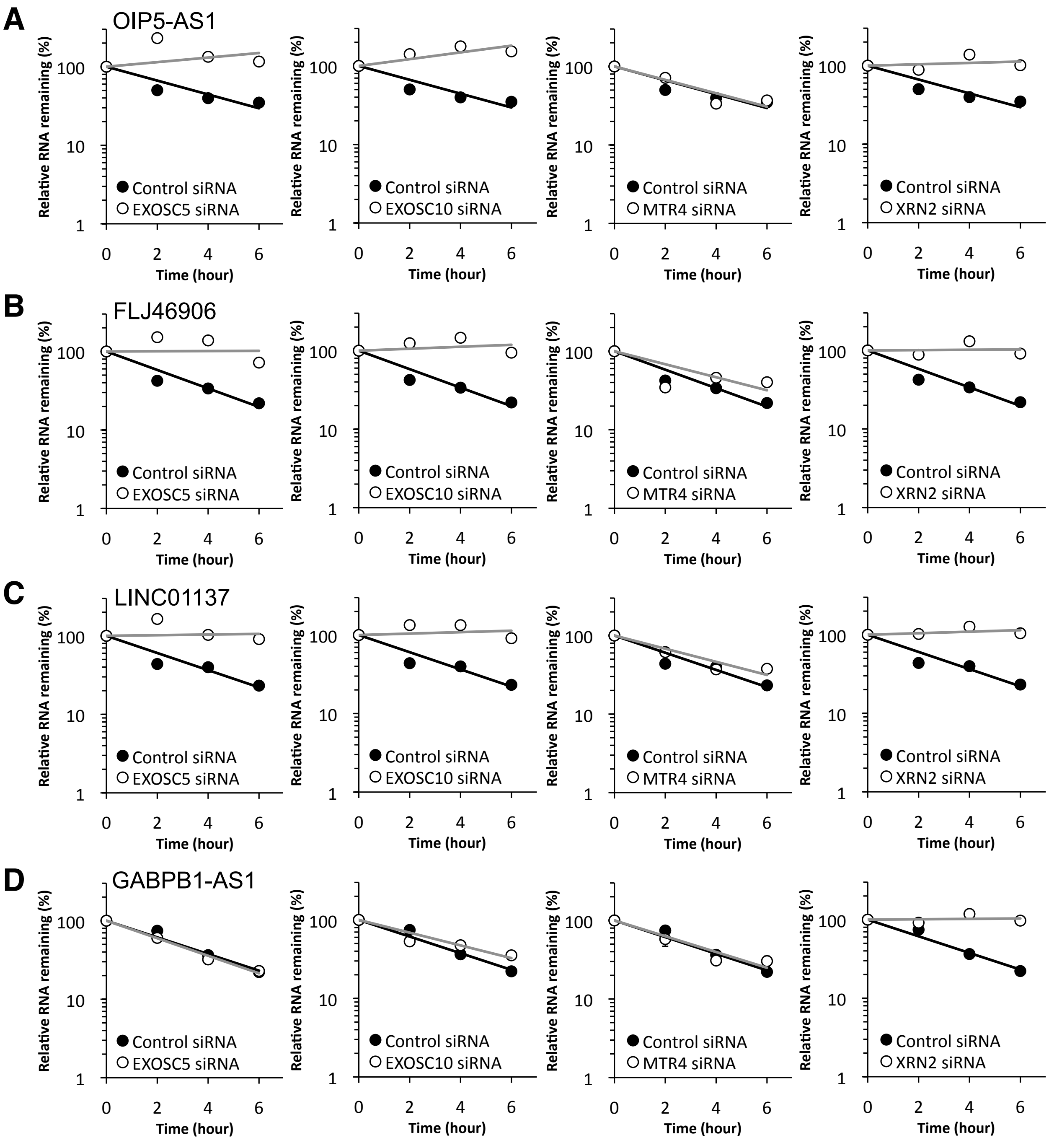

Figure S1. Knockdown of nuclear RNases and a helicase. The decay rates of (A) OIP5-AS1, (B) FLJ46906, (C) LINC00137, and (D) GABPB1-AS1 were determined in control cells with a control siRNA (solid circle and black line) and in treated cells with siRNAs targeting indicated nuclear RNases or a helicase (open circle and gray line). Values represent the mean ± SD obtained from three independent experiments.

Table S1. Primer pairs for RT-qPCR

Name	Sence sequence (5'-3')	Antisence sequence (5'-3')
GAPDH	GCACCGTCAAGGCTGAGAAC	TGGTGAAGACGCCAGTGGA
ACTB	CCAACCGCGAGAAGATGA	CCAGAGGCGTACAGGGATAG
HPRT1	TGACCTTGATTTATTTTGCATACC	CGAGCAAGACGTTCAGTCCT
PGK1	CTGTGGCTTCTGGCATACCT	CGAGTGACAGCCTCAGCATA
MIR22HG	CGGACGCAGTGATTTGCT	GCTTTAGCTGGGTCAGGACA
LINC-PINT	GGGATAATTTGCCATCTGGA	CCGTTTCTTCCATTTTCCTCT
KMT2E-AS1	CAGCCAAAATCTGGCCTACT	GTCCAGCAATCATTTTCTCGT
LINC00667	AGTTTGCGCCTTTTGGTCT	GGCCATGTGCAAAGGATTT
HCG18	CTGCCTTCTAGGGGCTCACT	ACATGCTCCACCAACTTTCA
UBA6-AS1	CTGAAATCAGAGCCTGCACA	TCAAGGACCTGGAAATGACC
LINC00662	GTTTGATTTCTCGCAGACCAG	GCGAGGTCTAACCCAGGTG
GABPB-AS1	AGGGAAAGAAAATATGCCATTTCTA	ATCATTCCGCCGCTTTCT
LINC01184	GCAATTATCACGGGAAACCTAT	AAATCTTATCTGCTTCCCTATTTGTAA
TTN-AS1	TCCTTAGGCATCACCTAGCC	GATGGAGGAAGTAGAGTCATTGG
LINC00473_v1	TATGCGCGTCAGCATACTTT	TGTCCTGTGCCTCCCTGT
LINC00473_v2	TATGCGCGTCAGCATACTTT	TCTCCCAAAGCACAACGAG
FAM222A-AS1	CAACATGGAAATGGAGACCA	CTTCCGGGATCCCAGTGT
CYTOR	CGTGCCTGTCTTCAGATCTTC	TCATCTCCCAGTTATTCAAGGAG
MIR4435-2HG_v1	CAGATCTTCACAGCACAGTTCC	TGCTGATCCACTTTGCTTGT
MIR4435-2HG_v2	CACCAGCCTCTCCCTGAA	TTCGATCAAGTGTGTCATAGAGC
IDI2-AS1	GTGTTAAACAAGACAACGCTGAA	AAGAGCGCTGGAAAAACCTT
SNHG15	GCAACTCCTTTGCAAGATGC	CTCAAGGAGGGACCTCAGC
ZFP91-CNTF	TTGTTCATACTTGGCGGTGA	GGCGGGCCTAATCATTTT
OIP5-AS1	GATTTCTGCTCACTGCAGTCTCT	CCTAGCTACTTGGGAGGCTGA
KMT2E-AS1	CACCCTCCGAAAGCGATAC	GAGAGGAACGGGGAGACG
EBLN3P	TTTGAGGACCGAGTAGTCCTG	AGCTCCCGAAATTTTGGATT
LINC01137	GGGGGAAGTTGTGTAACCTCT	CTCCAAGGGGCTTCGTTC
PVT1	TGAGAACTGTCCTTACGTGACC	AGAGCACCAAGACTGGCTCT
FLJ46906	GTGACCTCCCAGGCACAG	GTGAGAAGGGCTCCTCCAG
PP7080	ACAGGCCCCCAGTTTGTC	GAAGGCAGCTTTGCTCCTC
EXOSC5	GATCCTACATCCAAGCAAGAAAA	CAGCAGCTTCCGTTCCAC
EXOSC10	GCTGATTTCATCCATCAGCA	TCATATTGATAAGGATGTGCAAACA
MTR4	GAAGGGTGTACACATGAGGTTG	TCCAACTCGTGGTTTAAGTGG
XRN2	GAAGCCATTCTTCCAGATCAA	CTGTGTCAGATCCCTTAACATAGG

Table S2. siRNAs used for knockdown

Name	Sence sequence (5'-3')	Antisence sequence (5'-3')
Control siRNA	GTACCTGACTAGTCGCAGAAG	TCTGCGACTAGTCAGGTACGG
EXOSC5 siRNA	CAACACGUCUUCCGUUUCUdTdT	AGAAACGGAAGACGUGUUGdTdT
EXOSC5 siRNA-2	GCAAAGAGAUUUUCAACAAdTdT	UUGUUGAAAAUCUCUUUGCdTdT
EXOSC10 siRNA	GCUGCAGCAGAACAGGCCAdTdT	UGGCCUGUUCUGCUGCAGCdTdT
EXOSC10 siRNA-2	AAGAUUUUAGGAAGAAAUGGU	CAUUUCUUCCUAAAAUCUUCA
MTR4 siRNA	GAGUCAAUAACUGAAGACUdTdT	AGUCUUCAGUUAUUGACUCdTdT
MTR4 siRNA-2	GAGAGGCCAUUCAGUGUGUdTdT	ACACACUGAAUGGCCUCUCdTdT
XRN2 siRNA	GUCCUUCGAUAUCUCCUAAUA	UUAGGAGAUAUCGAAGGACUA
XRN2 siRNA-2	CAUCGUUAGAGAUUAGGGAAA	UCCCUAAUCUCUAACGAUGGC
